# Shifting Paradigms in Pediatric Cholelithiasis: A 13-Year Retrospective Study of the Clinicodemographic Profile, Risk Factors, and Outcomes

**DOI:** 10.7759/cureus.114160

**Published:** 2026-08-08

**Authors:** Varun Mahajan, Sanjay K Bhasin, Sunita Kumari, Chandla Nikita Kishore Singh, Vivek Bhagat, Sunny Babber

**Affiliations:** 1 Surgery, Government Medical College, Jammu, Jammu, IND; 2 Physiology, All India Institute of Medical Sciences, Vijaypur, Jammu, Jammu, IND; 3 Anesthesia and Critical Care, Government Medical College, Jammu, Jammu, IND; 4 Community Medicine, Acharya Shri Chander College of Medical Sciences and Hospital, Jammu, IND

**Keywords:** childhood obesity, gallstones, hemolytic disorders, laparoscopic cholecystectomy, pediatric cholelithiasis, risk factors, ultrasonography

## Abstract

Background: Pediatric cholelithiasis is becoming a more common disease all over the world, and its epidemiology is changing, with a decrease in the incidence of hemolytic causes and a rise in idiopathic and metabolic risk factors. However, long-term data is still limited in the Indian subcontinent. The aim of the present study was to assess the clinicodemographic profile, risk factors, presentation, management, and outcome of cholelithiasis in the pediatric age group in a tertiary care teaching hospital in North India for 13 years.

Materials and methods: This was a retrospective observational study of 380 children <15 years who were diagnosed with ultrasonographically confirmed cholelithiasis and treated from January 2013 to December 2025. Hospital records were used to collect demographic data, clinical features, risk factors, ultrasonographic data, operative data, histopathological data, postoperative recovery, and complications. Descriptive statistics included mean ± standard deviation (SD) for continuous variables and frequencies and percentages for categorical variables. Multivariable binary logistic regression was used to identify independent predictors of postoperative complications; results are presented as adjusted odds ratio (AOR) and 95% confidence interval (CI).

Results: The age group 11-14 years was the largest (212, 55.8%), and males had the highest prevalence (248, 65.3%). Of the patients, family history (70, 18.4%) and prior antibiotic use (56, 14.7%) were the most frequently recognized risk factors, with almost half (182, 47.9%) having no known association. The most common type of stones was mixed gallstones (246, 64.7%). The most frequent presenting symptom was vague upper abdominal pain (228, 60.0%), and the most common finding on ultrasonography was multiple gallstones (220, 57.9%). The success rate for cholecystectomy was 368 (96.8%), and the number of patients that had to be converted was 8 (2.1%), which was low, and postoperative recovery was excellent. Overall, 308 (81.0%) of patients had no postoperative complications. Multivariable analysis revealed that overweight/obesity, hemolytic disorders, multiple gallstones, intraoperative adhesions, mucocele/empyema, and operative duration >30 minutes were found to be independent factors associated with postoperative complications (AOR > 2, p < 0.05). Temporal trend analysis demonstrated progressive increases in idiopathic and obesity-associated cholelithiasis, accompanied by a decline in hemolytic disorder-associated disease and increasing utilization of laparoscopic cholecystectomy throughout the study period.

Conclusions: This 13-year study indicates that there is a definite epidemiological shift in the etiology of pediatric cholelithiasis, with hemolytic disease and idiopathic disease giving way to obesity-associated gallstones. Laparoscopic cholecystectomy was found to have excellent perioperative results, low morbidity, and quick recovery after surgery, suggesting it as the preferred surgical approach for symptomatic gallstones in children. These findings highlight the increasing role metabolic and lifestyle-related factors play in childhood gallstone disease and the importance of recognizing at-risk children early and of conducting multi-center prospective studies to further define the changing epidemiology of gallstones in children and improve management strategies.

## Introduction

Gallstone disease is one of the most common disorders affecting the hepatobiliary system and remains a significant cause of morbidity worldwide. It affects millions of individuals annually and accounts for a substantial number of hospital admissions and surgical procedures, particularly cholecystectomies. Although traditionally considered a disease of adults, especially middle-aged women, gallstone disease is increasingly being recognized in the pediatric population. The growing burden of pediatric cholelithiasis has attracted considerable attention because of its changing epidemiology, diverse clinical presentation, and evolving management strategies [1]. Historically, gallstones in children were regarded as rare and were primarily associated with hemolytic disorders such as sickle cell disease, hereditary spherocytosis, and thalassemia. Earlier studies suggested that most pediatric cases occurred secondary to underlying hematological abnormalities or congenital hepatobiliary disorders. However, subsequent investigations demonstrated that gallstone disease in children may occur in the absence of traditional risk factors and can present across a broad spectrum of age groups and clinical settings [2-5]. Indications for surgical management of pediatric gallstone disease have also evolved considerably over time. Earlier reports described cholecystectomy as an uncommon procedure in children, whereas contemporary pediatric surgical practice has witnessed a progressive increase in the number of cholecystectomies performed for symptomatic gallstones and related complications.

The expanding role of minimally invasive surgery has further contributed to improved outcomes and wider acceptance of surgical treatment in pediatric patients [6,7]. Another important factor contributing to the increased recognition of pediatric cholelithiasis is the widespread availability of ultrasonography. As a safe, non-invasive, readily accessible, and highly accurate imaging modality, ultrasonography has become the investigation of choice for diagnosing gallstones in children. Improvements in imaging technology have facilitated earlier diagnosis and have resulted in the detection of many asymptomatic and atypical cases that might previously have remained unrecognized [8]. Recent literature suggests that the distribution of etiological and risk-factor profiles in pediatric cholelithiasis is changing. While hemolytic disorders continue to play an important role, an increasing proportion of children now present with idiopathic or non-hemolytic gallstones. Contemporary studies have highlighted a growing contribution of genetic, metabolic, environmental, and lifestyle-related factors to gallstone formation. This shift has led many authors to propose that pediatric gallstone disease should no longer be viewed solely as a complication of hematological disorders but rather as a multifactorial condition with complex pathophysiological mechanisms [9]. Among the emerging risk factors, childhood obesity has gained particular attention. The global rise in obesity has been accompanied by increasing rates of insulin resistance, dyslipidemia, and metabolic syndrome among children and adolescents. These metabolic abnormalities contribute to cholesterol supersaturation of bile, impaired gallbladder motility, and subsequent gallstone formation. Several recent studies have reported a strong association between obesity and pediatric cholelithiasis, emphasizing the growing importance of metabolic health in the pathogenesis of the disease [10]. Current management strategies for pediatric gallstone disease continue to evolve as new evidence emerges. While observation may be appropriate for selected asymptomatic patients, laparoscopic cholecystectomy remains the treatment of choice for symptomatic disease and its complications. Recent guidelines and clinical studies support minimally invasive surgery because of its excellent safety profile, shorter hospital stay, reduced postoperative pain, rapid recovery, and favorable long-term outcomes [11]. Population-based studies have demonstrated a steady increase in hospitalization rates and healthcare utilization related to pediatric gallstone disease. These findings suggest that pediatric cholelithiasis is becoming an increasingly important clinical and public health concern [12]. Furthermore, advances in surgical techniques and perioperative care have resulted in excellent outcomes, with low complication rates and high success rates reported from specialized pediatric surgical centers [13]. Recent investigations have provided valuable insights into the clinical characteristics of pediatric cholelithiasis, highlighting considerable variability in age distribution, symptomatology, risk factors, and stone composition. Multiple studies have reported that abdominal pain remains the most common presenting symptom, although a substantial proportion of patients may remain asymptomatic and are diagnosed incidentally during imaging performed for unrelated conditions [14].

Laparoscopic cholecystectomy has emerged as the gold standard treatment for symptomatic pediatric gallstone disease and has largely replaced conventional open surgery. Contemporary evidence demonstrates excellent postoperative outcomes, minimal morbidity, and rapid return to routine activities following laparoscopic intervention [15]. Special populations, including infants, premature children, and those with complex medical conditions, continue to present unique diagnostic and therapeutic challenges requiring individualized management strategies [16]. Recent review articles have emphasized the need for ongoing research into the changing distribution of etiological and risk-factor profiles in pediatric cholelithiasis, particularly in developing countries where data remain limited. Growing evidence suggests that dietary transitions, urbanization, changing lifestyle patterns, and increasing obesity rates may further contribute to the future burden of gallstone disease among children and adolescents [17,18]. Despite increasing recognition of pediatric cholelithiasis worldwide, comprehensive data from the Indian subcontinent remain relatively scarce. Moreover, significant regional variations exist regarding demographic characteristics, associated risk factors, clinical presentation, and treatment outcomes. Therefore, this study was undertaken to comprehensively evaluate pediatric cholelithiasis over a 13-year period at a tertiary care teaching hospital in North India. The primary objective was to describe the clinicodemographic characteristics, associated risk factors, clinical presentation, ultrasonographic findings, surgical management, histopathological features, and postoperative outcomes of children with cholelithiasis. The secondary objectives were to evaluate temporal changes in the distribution of associated risk factors during the study period and to identify factors associated with postoperative complications following cholecystectomy.

## Materials and methods

This retrospective observational study was carried out in the postgraduate Department of Surgery, Government Medical College and Associated Hospitals, Jammu, a tertiary care referral hospital serving the people of Jammu and its adjoining areas of North India. The medical records of children who were diagnosed with cholelithiasis from January 2013 to December 2025 were retrieved. The eligible patients were identified using a consecutive sampling method. A total of 530 pediatric patients with suspected or ultrasonographically confirmed cholelithiasis. Of these, 380 children aged 1-14 years with ultrasonographically confirmed cholelithiasis data and complete demographic, clinical, radiological, operative, histopathological, and follow-up records were included in the final analysis. Of these, 150 patients were excluded because of incomplete medical records, uncertain diagnosis, referral before completion of evaluation or treatment, or missing operative or follow-up information. No formal sample size calculation was done as all eligible patients were treated during the 13-year study period.

Data were collected from hospital records, operative registers, discharge summaries, radiology records and histopathology reports with a predesigned proforma. The demographic variables considered were age, sex, residence, socioeconomic status and dietary pattern. A Modified Kuppuswamy Socioeconomic Scale was used as a classification of socioeconomic status [19]. Clinical parameters were associated symptoms, symptom duration, family history of gallstones, previous antibiotic therapy, prematurity, hemolytic disorders, hypertriglyceridemia, other comorbidities, and anthropometric parameters. Patients were classified on the basis of the documented risk factors. Individuals who did not have any known recognized risk factor were categorized as no known recognized risk factor. Patients may have multiple risk factors; so these categories were not mutually exclusive. Weight and height were recorded at admission, and body mass index (BMI) was calculated. Nutritional status was assessed using BMI-for-age according to the World Health Organization (WHO) Growth Standards and Growth Reference. Radiological parameters consisted of gallstones, biliary sludge, gallbladder polyps, common bile duct stones, and other biliary tract abnormalities. The following operative variables were recorded: type of surgical procedure, operative duration, intra-operative findings, conversion to open surgery, and intra-operative complications. Conversion to open surgery was performed when laparoscopic completion was considered unsafe because of operative findings or technical difficulty, according to the operating surgeon's judgment. When clinically indicated or when the treating surgeon judged that it was clinically indicated, cholecystectomy was performed in the patients who were symptomatic. Asymptomatic patients were selected for surgery after individual clinical assessment. Histopathological changes were documented as normal gallbladder mucosa, chronic cholecystitis, acute cholecystitis, xanthogranulomatous cholecystitis, or any other pathological changes. Since the gallstone composition was not routinely analyzed biochemically, the morphologies of gallstones were recorded from operative findings and gross pathological descriptions documented in the medical records (mixed, pigment, or cholesterol). Complications, hospital stay, time to ambulation, bowel function, and time to return to normal daily activity were the postoperative outcomes studied. Postoperative complications were recorded during the available follow-up period documented in the hospital records. Owing to the retrospective nature of the study, only complications recorded during routine inpatient care and documented follow-up visits were included in the analysis.

Ethical approval

The study protocol has been approved by the Institutional Ethics Committee, Government Medical College, Jammu (Approval No. IEC/GMC/2025/1373, dated 03 July 2025). The Institutional Ethics Committee waived the informed consent requirement as the study was retrospective and based on the records. Patients' confidentiality was ensured throughout the study according to institutional ethical norms.

Statistical analysis

Data were entered into Microsoft Excel (Microsoft Corp., Redmond, WA, USA) and analyzed by IBM SPSS Statistics for Windows, Version 21 (Released 2012; IBM Corp., Armonk, New York, United States). Normality of continuous variables was determined with the Shapiro-Wilk test. Continuous variables are presented as mean and standard deviation (SD), and categorical variables are presented as frequencies and percentages. Multivariable binary logistic regression was used to determine factors associated with postoperative complications. Odds ratios (ORs) were adjusted and presented as adjusted ORs (AORs) with 95% confidence intervals (CIs). Records were discarded prior to analysis due to lack of information. All statistical tests were carried out as two-tailed tests, and a value of <0.05 was designated as the statistically significant level.

## Results

A total of 380 pediatric patients with ultrasonographically confirmed cholelithiasis who fulfilled the eligibility criteria were included in the study, with a mean age of 12.34 ± 1.54 years.

Table 1 shows that the majority of patients belonged to the 11-14-year age group (212, 55.8%), followed by children aged 5-10 years (130, 34.2%), whereas only 38 (10.0%) patients were younger than five years. A clear male predominance was observed, with 248 (65.3%) males and 132 (34.7%) females, resulting in a male-to-female ratio of approximately 1.9:1. Most patients originated from rural areas (286, 75.3%), while 94 (24.7%) resided in urban areas. Regarding socioeconomic status, 254 (66.8%) belonged to the lower socioeconomic group and 126 (33.2%) to the middle socioeconomic group. A mixed dietary pattern was reported by 268 (70.5%) patients, whereas 112 (29.5%) followed a vegetarian diet. Nearly half of the patients (182, 47.9%) had no identifiable risk factor. Among recognized risk factors, family history of gallstone disease (70, 18.4%) was the most common, followed by previous antibiotic therapy (56, 14.7%), overweight/obesity (38, 10.0%), hemolytic disorders (20, 5.3%), history of prematurity (20, 5.3%), and hypertriglyceridemia (18, 4.7%). These findings indicate that idiopathic cholelithiasis constituted the largest subgroup in the present study.

**Table 1:** Demographic characteristics and risk factors of pediatric patients with cholelithiasis (N = 380)

Variable	Frequency (n)	Percentage (%)
Age group (years)		
1-4	38	10.0
5-10	130	34.2
11-14	212	55.8
Gender		
Male	248	65.3
Female	132	34.7
Residence		
Rural	286	75.3
Urban	94	24.7
Socioeconomic status		
Lower	254	66.8
Middle	126	33.2
Dietary pattern		
Vegetarian	112	29.5
Mixed diet	268	70.5
Risk factors		
Family history of gallstones	70	18.4
Previous antibiotic therapy	56	14.7
Overweight/obesity	38	10.0
Hemolytic disorder	20	5.3
History of prematurity	20	5.3
Hypertriglyceridemia	18	4.7
No identifiable risk factor	182	47.9

**Figure 1:**
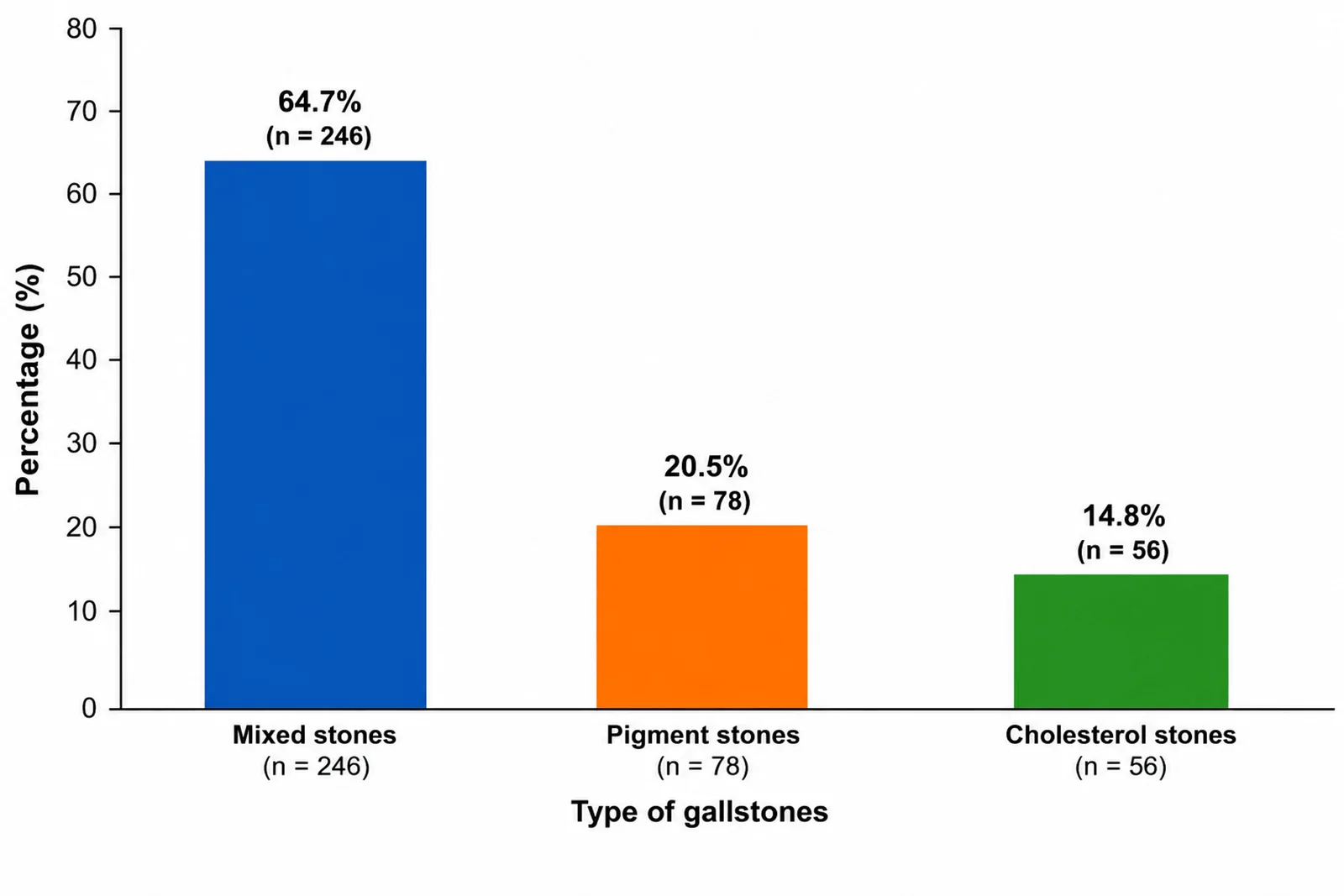
Distribution of gallstone morphology among 380 pediatric patients with cholelithiasis

Figure 1 illustrates the distribution of gallstone morphology among the 380 pediatric patients included in the study. Mixed gallstones constituted the predominant stone type, accounting for 246 (64.7%) cases. Pigment stones were identified in 78 (20.5%) patients, while cholesterol stones were observed in 56 (14.8%) patients. Thus, nearly two-thirds of all gallstones were of mixed composition, whereas cholesterol stones represented the least common subtype in the study population.

**Figure 2:**
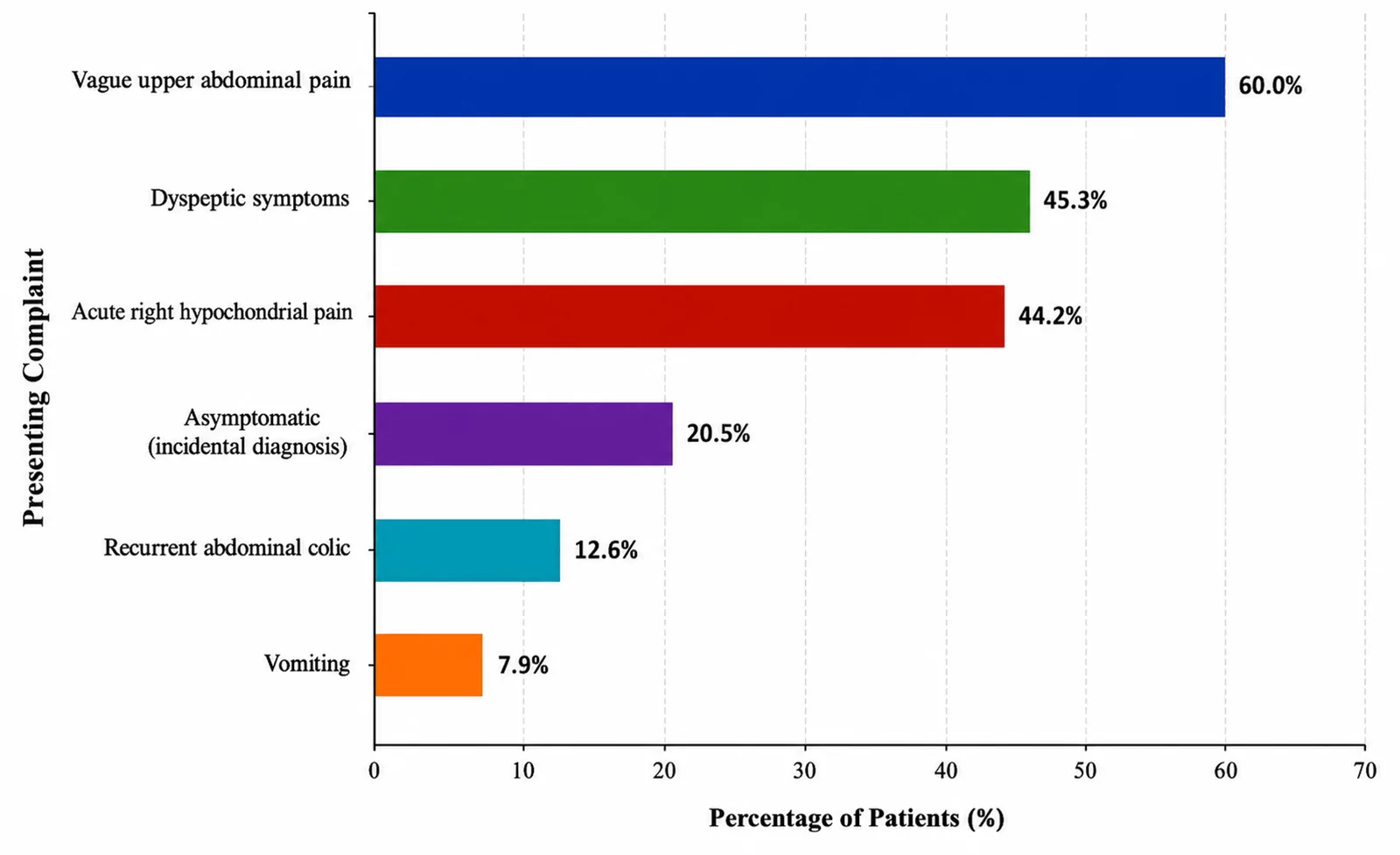
Frequency of clinical presentations among 380 pediatric patients with cholelithiasis

Figure 2 depicts the clinical manifestations of pediatric cholelithiasis. The most common presenting symptom/complaint was vague upper abdominal pain, reported by 228 (60.0%) patients. Dyspeptic symptoms and acute right hypochondrial pain were present in 172 (45.3%) and 168 (44.2%) patients, respectively. Asymptomatic gallstones, detected incidentally during ultrasonographic evaluation, were observed in 78 (20.5%) patients. Recurrent abdominal colic was reported by 48 (12.6%), while vomiting occurred in 30 (7.9%) patients. Since several patients presented with more than one symptom, the percentages exceed 100%.

**Figure 3:**
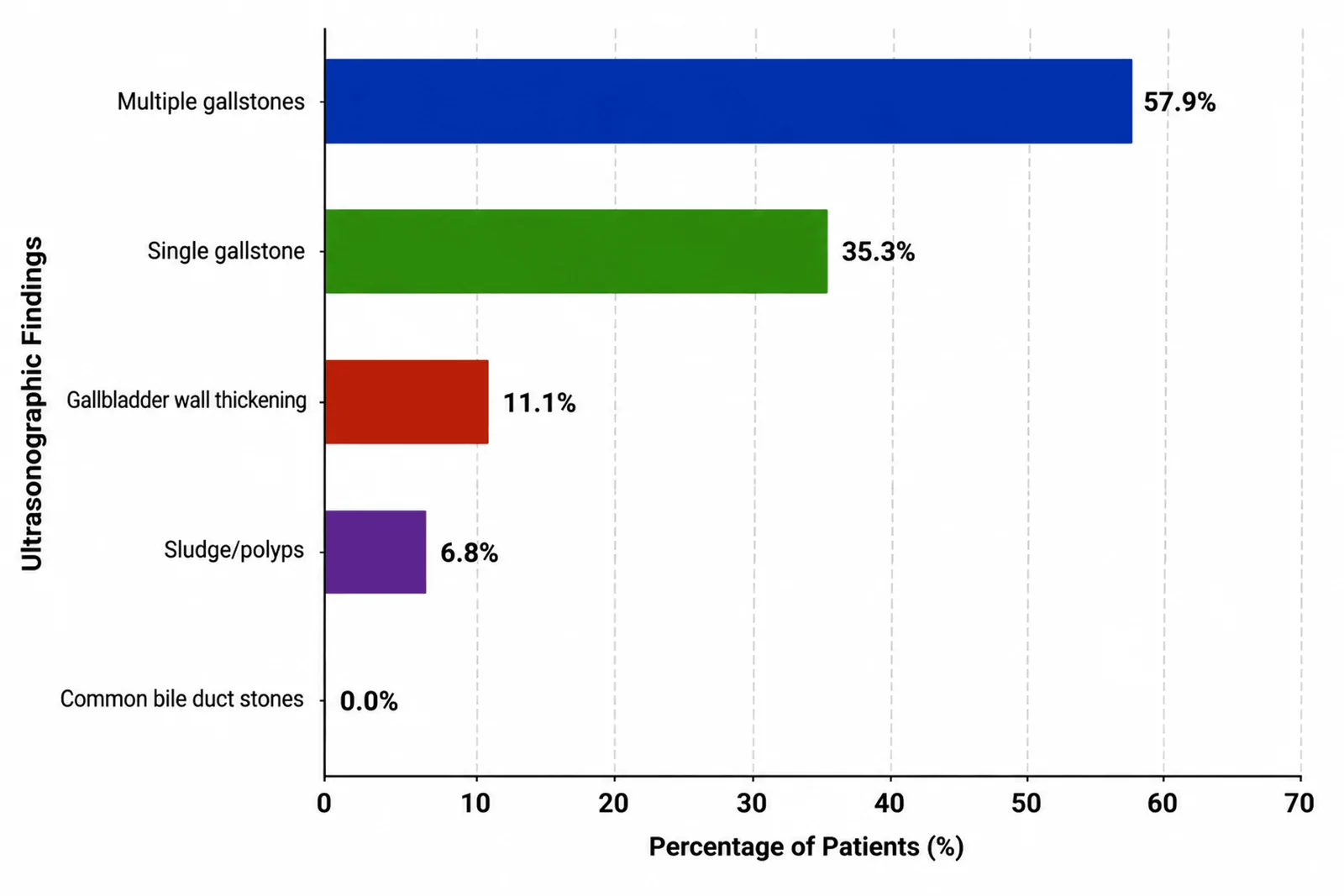
Ultrasonographic characteristics of pediatric cholelithiasis among 380 patients

Figure 3 summarizes the ultrasonographic findings in the study population. Multiple gallstones were the most frequent finding, observed in 220 (57.9%) patients, whereas single gallstones were detected in 134 (35.3%) patients. Associated gallbladder wall thickening was present in 42 (11.1%) patients, and gallbladder sludge or polyps were identified in 26 (6.8%) patients. No patient demonstrated common bile duct stones on ultrasonographic examination. These findings highlight the high diagnostic yield of ultrasonography in detecting both gallstones and associated gallbladder abnormalities.

Table 2 summarizes the operative findings and surgical management. Adhesions were the most common intraoperative finding, observed in 70 (18.4%) patients. Mucocele and empyema of the gallbladder were identified in 16 (4.2%) and six (1.6%) patients, respectively. With regard to operative management, laparoscopic cholecystectomy was successfully performed in 368 (96.8%) patients, confirming its role as the preferred surgical approach. Only eight (2.1%) patients required conversion to open surgery, while four (1.1%) underwent mini-cholecystectomy. Analysis of gallstone morphology revealed that mixed stones were the most common (246, 64.7%), followed by pigment stones (78, 20.5%) and cholesterol stones (56, 14.8%). These findings demonstrate the predominance of minimally invasive surgery and mixed gallstone morphology in the present cohort.

**Table 2:** Intraoperative findings, surgical procedures, and gallstone characteristics (N = 380)

Variable	Frequency (n)	Percentage (%)
Intraoperative findings		
Adhesions	70	18.4
Mucocele	16	4.2
Empyema	6	1.6
Surgical procedure		
Laparoscopic cholecystectomy	368	96.8
Conversion to open surgery	8	2.1
Mini-cholecystectomy	4	1.1
Gallstone morphology		
Mixed stones	246	64.7
Pigment stones	78	20.5
Cholesterol stones	56	14.8

Table 3 presents that postoperative recovery was uneventful in the majority of patients. Overall, 308 (81.0%) patients experienced no postoperative complications. Among those with complications, shoulder tip pain was the most common (30, 7.9%), followed by nausea and vomiting (20, 5.3%) and wound infection (20, 5.3%). Biliary peritonitis was uncommon and occurred in only two (0.5%) patients. Histopathological examination demonstrated normal gallbladder mucosa in 224 (58.9%) patients. Chronic cholecystitis was identified in 96 (25.3%), mild acute cholecystitis in 48 (12.6%), and xanthogranulomatous cholecystitis in 12 (3.2%) patients. These findings indicate that advanced inflammatory pathology was relatively uncommon in this study population.

**Table 3:** Postoperative outcomes and histopathological findings (N = 380)

Parameter	Frequency (n)	Percentage (%)
Postoperative complications		
Shoulder tip pain	30	7.9
Nausea/vomiting	20	5.3
Wound infection	20	5.3
Biliary peritonitis	2	0.5
No complications	308	81.0
Histopathological findings		
Normal mucosa	224	58.9
Chronic cholecystitis	96	25.3
Mild acute cholecystitis	48	12.6
Xanthogranulomatous cholecystitis	12	3.2

Table 4 summarizes the postoperative recovery profile following cholecystectomy. The mean operative time was 29.0 ± 6.4 minutes, while the mean hospital stay was 1.9 ± 0.8 days, indicating efficient perioperative management. Patients resumed routine daily activities after a mean of 4.0 ± 1.2 days. Early postoperative recovery was satisfactory, with 298 (78.4%) patients becoming ambulatory on the first postoperative day, while 302 (79.5%) passed flatus within the same period, indicating early restoration of gastrointestinal function. Overall, these findings demonstrate rapid postoperative recovery following laparoscopic cholecystectomy.

**Table 4:** Postoperative recovery profile (N = 380)

Recovery Variable	Value
Mean operative time (minutes)	29.0 ± 6.4
Mean hospital stay (days)	1.9 ± 0.8
Return to routine activity (days)	4.0 ± 1.2
Ambulatory on postoperative day 1	298 (78.4%)
Passed flatus on postoperative day 1	302 (79.5%)

**Figure 4:**
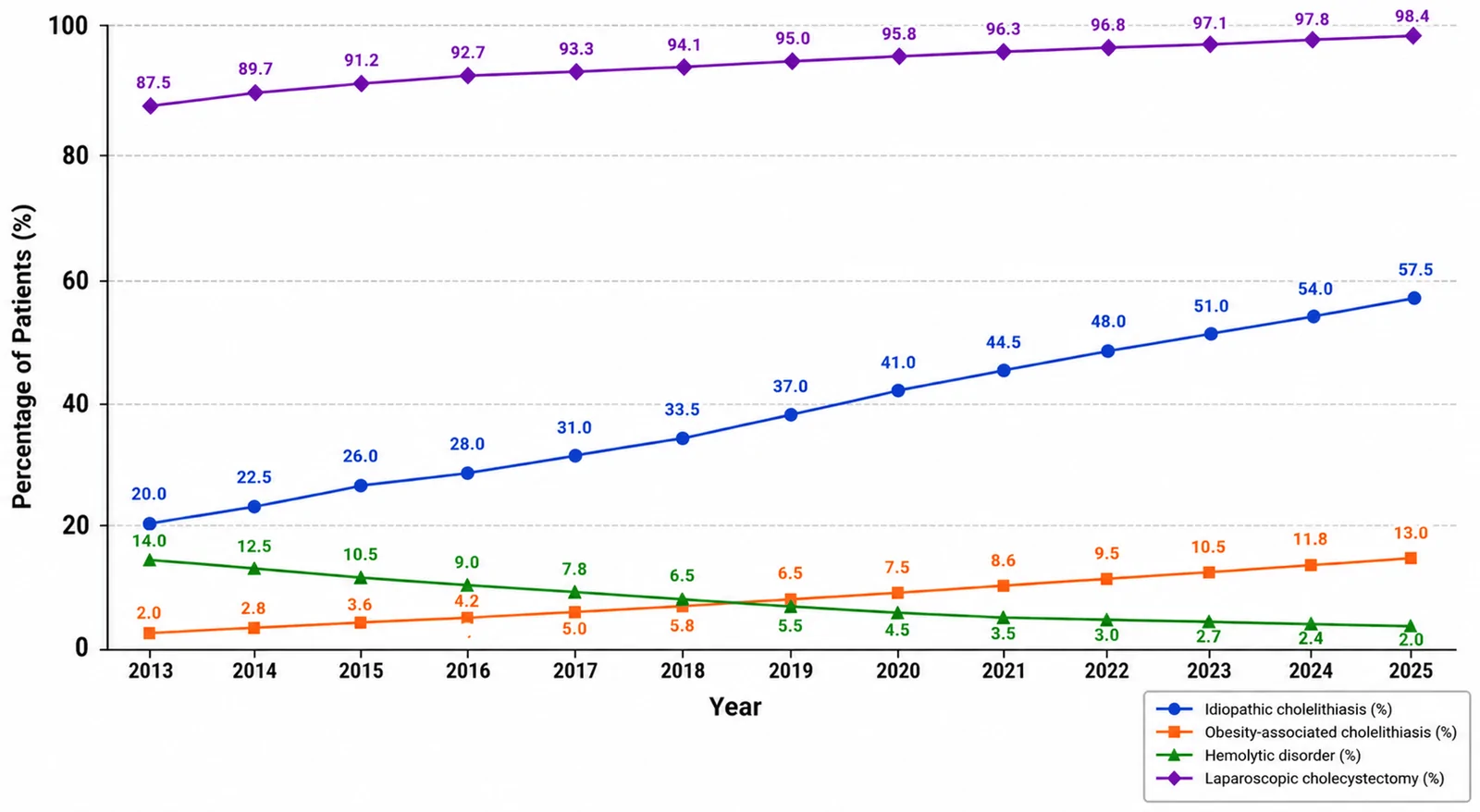
Temporal trends in pediatric cholelithiasis and surgical management (2013-2025)

Figure 4 demonstrates the changing epidemiological trends in pediatric cholelithiasis over the 13-year study period. The proportion of idiopathic cholelithiasis increased steadily from 20.0% in 2013 to 57.5% in 2025, indicating a progressive rise in cases without identifiable traditional risk factors. Similarly, obesity-associated cholelithiasis showed a consistent upward trend, increasing from 2.0% to 13.0% during the study period. In contrast, the proportion of patients with hemolytic disorder-associated cholelithiasis declined progressively from 14.0% in 2013 to 2.0% in 2025. Over the same period, the utilization of laparoscopic cholecystectomy increased from 87.5% to 98.4%, reflecting the growing adoption of minimally invasive surgery as the standard treatment modality for pediatric gallstone disease.

Table 5 presents the multivariable binary logistic regression analysis identifying factors associated with postoperative complications. Overweight/obesity was associated with a significantly increased risk of postoperative complications (AOR 2.63; 95% CI 1.31-5.27; p = 0.006). Similarly, hemolytic disorders significantly increased the likelihood of complications (AOR 2.18; 95% CI 1.01-4.71; p = 0.047). Patients with multiple gallstones had more than twice the odds of developing postoperative complications (AOR 2.34; 95% CI 1.29-4.26; p = 0.005). Among intraoperative findings, adhesions emerged as the strongest independent predictor (AOR 3.82; 95% CI 2.01-7.25; p < 0.001), while the presence of mucocele or empyema also significantly increased the risk (AOR 2.79; 95% CI 1.14-6.83; p = 0.024). An operative duration exceeding 30 minutes was independently associated with postoperative complications (AOR 2.41; 95% CI 1.37-4.22; p = 0.002). In contrast, age, sex, rural residence, dietary pattern, family history of gallstones, previous antibiotic therapy, hypertriglyceridemia, and the surgical approach (laparoscopic versus open/mini-open cholecystectomy) were not statistically significant independent predictors of postoperative complications (all p > 0.05). These findings suggest that metabolic factors and intraoperative disease severity had a greater influence on postoperative outcomes than demographic characteristics.

**Table 5:** Multivariable binary logistic regression analysis identifying independent predictors of postoperative complications among pediatric patients with cholelithiasis (N = 380) β: regression coefficient; SE: standard error; CI: confidence interval; p < 0.05 is statistically significant.

Variable	β Coefficient	SE	Adjusted Odds Ratio (AOR)	95% CI	P value
Age ≥11 years	0.39	0.28	1.48	0.84-2.61	0.173
Male sex	0.11	0.28	1.12	0.65-1.95	0.682
Rural residence	0.15	0.30	1.16	0.64-2.09	0.623
Mixed diet	0.19	0.31	1.21	0.66-2.23	0.536
Family history of gallstones	0.38	0.31	1.46	0.80-2.67	0.220
Previous antibiotic therapy	0.31	0.34	1.37	0.70-2.67	0.367
Overweight/obesity	0.97	0.35	2.63	1.31-5.27	0.006
Hemolytic disorder	0.78	0.39	2.18	1.01-4.71	0.047
Hypertriglyceridemia	0.42	0.47	1.52	0.61-3.78	0.366
Multiple gallstones	0.85	0.30	2.34	1.29-4.26	0.005
Adhesions	1.34	0.33	3.82	2.01-7.25	<0.001
Mucocele/empyema	1.03	0.46	2.79	1.14-6.83	0.024
Operative time >30 minutes	0.88	0.29	2.41	1.37-4.22	0.002
Laparoscopic cholecystectomy (vs. open/mini-open)	−0.58	0.42	0.56	0.24-1.29	0.173

## Discussion

The present retrospective study evaluated the clinicodemographic profile, risk factors, clinical presentation, operative findings, and outcomes of 380 pediatric patients with cholelithiasis managed over a 13-year period at a tertiary care center in North India. The findings highlight the changing distribution of associated risk factors in pediatric gallstone disease and provide evidence supporting a gradual shift from traditional hemolytic etiologies toward predominantly idiopathic and metabolically associated disease. A notable observation in the present study was the predominance of adolescents aged 11-14 years, who constituted more than half of the study population. Similar age distributions have been reported by Bogue et al., Kirsaclioglu et al., and Todesco et al., who demonstrated that the incidence of pediatric cholelithiasis increases during adolescence, likely due to hormonal influences, dietary factors, and metabolic changes occurring during puberty [2,3,9]. Male predominance was observed in our cohort, a finding that has been reported in several pediatric studies, although gender distribution remains variable across different populations [2,14]. One of the most important findings of the present study was the demonstration of changing temporal trends over the 13-year study period. The proportion of idiopathic cases increased progressively, while gallstones associated with hemolytic disorders declined substantially. Similarly, obesity-associated cholelithiasis showed a gradual increase over time. These observations are consistent with recent literature suggesting that the pattern of associated risk factors in pediatric cholelithiasis is changing.

In our hospital-based cohort, the relative contribution of obesity-associated disease increased over time, whereas the proportion of hemolytic disorder-associated disease declined. These findings should be interpreted as changes within our study population rather than evidence of population-level epidemiological trends [8,9,18]. These findings reflect changing patterns of associated risk factors within our hospital-based cohort and may be influenced by changing lifestyle and nutritional factors. Nearly half of the patients had no identifiable risk factor, making this the largest risk-factor category in our cohort. Similar findings have been reported by Zdanowicz et al. and Svensson and Makin, who observed a growing proportion of children presenting without underlying hemolytic disease or congenital hepatobiliary abnormalities [8,18]. Among identifiable risk factors, family history represented the most frequent association, supporting previous evidence regarding the role of genetic susceptibility in gallstone formation. Frybova et al. reported similar findings and suggested that inherited alterations in cholesterol metabolism, bile composition, and gallbladder motility may contribute significantly to disease development [4]. Although obesity was present in only a minority of patients, its prevalence increased during the study period. Previous studies by Koebnick et al. and Fradin et al. have demonstrated a strong relationship between childhood obesity, insulin resistance, dyslipidemia, and cholesterol gallstone formation [20,21]. The increasing contribution of obesity observed in our study mirrors global trends and suggests that metabolic factors are becoming increasingly relevant in pediatric gallstone disease. The clinical presentation of pediatric cholelithiasis was frequently nonspecific. Vague upper abdominal pain was the most common presenting complaint, followed by dyspeptic symptoms and acute right hypochondrial pain. Similar symptom patterns have been reported by Stringer and Wesdorp et al., who emphasized that pediatric gallstone disease often lacks the classical presentation observed in adults [13,14]. More than one-fifth of patients in our study were asymptomatic and diagnosed incidentally during ultrasonographic examination, reflecting increased utilization of abdominal imaging and improved diagnostic capabilities [8,11].

Ultrasonography remained the primary diagnostic modality and demonstrated excellent utility in detecting gallstones. Multiple gallstones were more frequently observed than solitary stones, a finding comparable with previous reports [10,14]. The non-invasive nature, absence of radiation exposure, and high diagnostic accuracy of ultrasonography continue to make it the investigation of choice in pediatric patients. Laparoscopic cholecystectomy was the predominant treatment modality and was successfully completed in the vast majority of patients. The low conversion rate and favorable postoperative outcomes observed in our study are consistent with reports from Lee et al., St Peter et al., and Rescorla and Grosfeld, who demonstrated that laparoscopic cholecystectomy is a safe and effective treatment for symptomatic pediatric gallstone disease [7,15,16]. Advances in minimally invasive surgical techniques and perioperative care have contributed significantly to improved outcomes and reduced morbidity. Mixed stones constituted the most common gallstone type, followed by pigment and cholesterol stones. Similar observations have been reported by Frybova et al. and Della Corte et al., supporting the multifactorial etiology of pediatric cholelithiasis [4,5]. Histopathological examination predominantly revealed normal mucosa or chronic inflammatory changes, suggesting that many patients underwent surgical intervention before the development of advanced gallbladder pathology. Postoperative recovery was excellent, with short operative duration, minimal hospital stay, early ambulation, and low complication rates. The findings highlight the changing distribution of associated risk factors in pediatric gallstone disease. These findings are comparable to those reported in contemporary pediatric surgical series and further support the role of laparoscopic cholecystectomy as the gold-standard treatment for symptomatic pediatric gallstone disease [7,11,16]. The present study has several strengths, including a relatively large sample size, long study duration, and comprehensive assessment of demographic, clinical, radiological, operative, and histopathological parameters.

Limitations

There are some limitations of the present study. First, it is a retrospective study, which may have inherent information and selection bias due to the retrospective information obtained from medical records. In addition, the exclusion of 150 patients because of incomplete records or missing follow-up information may have introduced selection bias and should be considered while interpreting the findings. Second, this study is a single-center study performed at a tertiary care referral hospital and therefore the results may not be easily generalizable to other populations or healthcare settings. Third, there was insufficient long-term follow-up information to assess long-term postoperative complications, recurrence rates, and quality-of-life outcomes. Lastly, biochemical composition of gallstones was not performed routinely in all cases, making detailed examination of gallstone composition and its relation to the underlying etiological factors difficult. Despite these limitations, the present study includes a relatively large cohort with detailed demographic, clinical, radiological, operative, and histopathological data collected over a 13-year period. These findings provide useful information on the changing distribution of associated risk factors and the contemporary management of pediatric cholelithiasis. Further prospective multicenter studies with standardized follow-up and biochemical analysis of gallstones are required to validate these findings.

## Conclusions

This 13-year retrospective study showed changes in the pattern of risk factors among children with cholelithiasis treated at our tertiary care center. Children with no identifiable risk factor formed the largest group, while obesity-associated cholelithiasis became more common over time and the proportion of cases associated with hemolytic disorders gradually declined. Ultrasonography remained a reliable tool for diagnosis, and laparoscopic cholecystectomy was associated with favorable surgical outcomes, including low complication rates and early recovery. Our findings suggest that metabolic and lifestyle-related factors are playing an increasingly important role in pediatric gallstone disease. Early recognition of children at risk and timely surgical intervention, when indicated, may help improve clinical outcomes. As this was a single-center retrospective study, the findings should be interpreted with appropriate caution. Further prospective multicenter studies with larger patient populations and longer follow-up are needed to confirm these findings and to better understand the changing distribution of risk factors in pediatric cholelithiasis.
